# Performance Evaluation of a Biometric System Based on Acoustic Images

**DOI:** 10.3390/s111009499

**Published:** 2011-10-10

**Authors:** Alberto Izquierdo-Fuente, Lara del Val, María I. Jiménez, Juan J. Villacorta

**Affiliations:** Departamento de Teoría de la Señal y Comunicaciones e Ingeniería Telemática, Universidad de Valladolid, Paseo Belén 15, 47011, Valladolid, Spain; E-Mails: lara.val@tel.uva.es (L.V.); marjim@tel.uva.es (M.I.J.); juavil@tel.uva.es (J.V.)

**Keywords:** acoustic imaging, biometry, beamforming

## Abstract

An acoustic electronic scanning array for acquiring images from a person using a biometric application is developed. Based on pulse-echo techniques, multifrequency acoustic images are obtained for a set of positions of a person (front, front with arms outstretched, back and side). Two Uniform Linear Arrays (ULA) with 15 λ/2-equispaced sensors have been employed, using different spatial apertures in order to reduce sidelobe levels. Working frequencies have been designed on the basis of the main lobe width, the grating lobe levels and the frequency responses of people and sensors. For a case-study with 10 people, the acoustic profiles, formed by all images acquired, are evaluated and compared in a mean square error sense. Finally, system performance, using False Match Rate (FMR)/False Non-Match Rate (FNMR) parameters and the Receiver Operating Characteristic (ROC) curve, is evaluated. On the basis of the obtained results, this system could be used for biometric applications.

## 1. Introduction

Biometric identification [1–3] is a subject of active research, where new algorithms and sensors are being developed. The most widely used identification systems are based on fingerprints, hand geometry, retina, face, voice, vein, signature, etc. The fusion of information from multiple biometric systems is also improving the performance of identification and verification systems [4].

Radar-based systems require expensive hardware and can be unreliable due to the very low reflection intensity from humans. Acoustic imaging provides a simple and cheap sensor alternative that allows for very precise range and angular information. Specifically in the acoustic field, there are two accurate and reliable classification systems for targets:

1. Animal echolocation, performed by mammals such as bats, whales and dolphins, where Nature has developed specific waveforms for each type of task [5,6] such as the classification of different types of flowers [7].
2. Acoustic signatures used in passive sonar systems [8,9], which analyze the signal received by a target in the time-frequency domain.

There are few papers working on acoustic imaging in air for the detection of human beings. Moebus and Zoubir [10,11] worked with the ultrasonic band (50 kHz) using a 2D array and beamforming in reception. They analyzed solid objects (poles and a cuboid on a pedestal) in their first work and human images more recently. They showed that humans have a distinct acoustic signature and proposed to model the echoes from the reflection parts of objects in the scene by a Gaussian-Mixture-Model. Based on the parameters of this model, a detector could be designed to discriminate between persons and non-person objects.

In previous works, the authors of this paper have developed multisensor surveillance and tracking systems based on acoustic arrays and image sensors [12,13]. After an exhaustive search in the literature, we have not found any papers on acoustic imaging in air for biometric verification of humans. Consequently, we launched a new line of research to develop a novel biometric system, based on acoustic images acquired with electronic scanning arrays. Humans are acoustically scanned by an active system working from 6 to 12 kHz (audio band), that registers acoustic images. Based on these images, the system can identify people using a previously acquired database of acoustic images.

**Figure 1.**
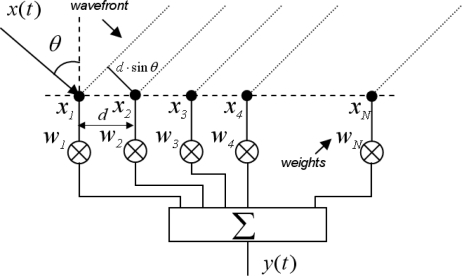
Structure of a beamformer.

Assuming a plane wave *x(t)* with a direction of arrival *θ,* and an array with *N* sensors separated a distance *d*, the signal received at each sensor *x_n_*, is a phase-shifted replica of *x(t)*. A beamformer combines linearly the signals *x_n_*, which are previously multiplied by complex weights *w_n_*, obtaining an output signal *y(t)*. Figure 1, shows the structure of a beamformer.

**Figure 2.**
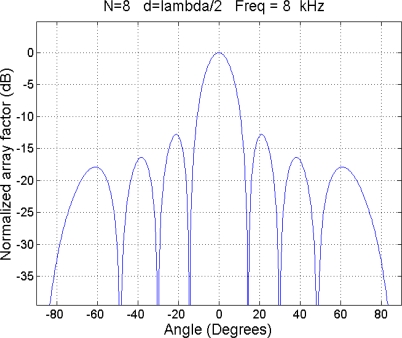
Beampattern for *θ* = 0°.

By means of the selection of the weights, it is possible to generate a narrow beam steered to a given direction, called steering angle, and therefore to implement an electronic scanning array [14,15]. The spatial response of a beamformer is called the array factor, and its graphical representation is the beampattern. Figure 2 shows a beampattern of an array with 8 λ/2-equispaced sensors, for a steering angle of 0°.

The proposed system uses beamforming, with a linear microphone array and a linear tweeter array, in transmission and reception, respectively. In this paper, Section 2 describes the system including the hardware architecture and the functional description. Section 3 designs the system parameters and characterizes the acoustic array sensor for these parameters. Section 4 describes the definition and extraction of acoustic profiles and Section 5 tests these images for biometry applications, defining a metric based on mean square error, and presents the obtained FMR/FNMR parameters and ROC curve. Finally, Section 6 presents our conclusions.

## 2. Description of the System

### 2.1. Functional Description

**Figure 3.**
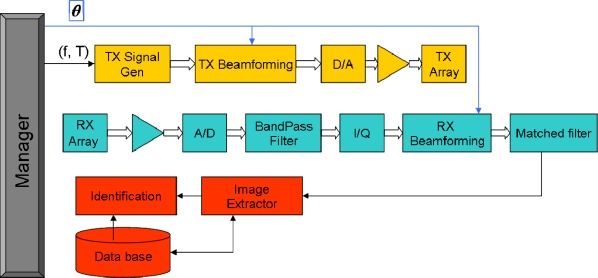
Block diagram.

Based on basic Radar/Sonar principles [17,18], an acoustic sound detection and ranging system for biometric identification is proposed, according to the block diagram in Figure 3.

The manager controls all subsystems, performing three main tasks: (i) person scanning and detection, (ii) acoustic images acquisition and (iii) person identification based on a database of acoustic images.

The following system parameters can be defined:

- A scanning area in azimuth: [*θ_min_ – θ_max_*]
- A scanning area in range: [*R_1_ – R_2_*]
- A collection of steering angles: *θ_1_, θ_2_ … θ_M_*
- Frequency *f* and pulse length *T*

For each steering angle, the system performs the following tasks:

- Transmission
  - For each sensor of the array, a sinusoidal pulse sequence with frequency *f*, phase *ϕ_i_* and length *T* is generated.
  - Transmission beamforming for steering angle *θ_i_* is done.
  - Sequences are sent to the D/A converter.
  - Signals are amplified and tweeters of the TX array are stimulated.
- Reception
  - Signals from microphones of RX array are preamplified.
  - The A/D converter samples the preamplified signals.
  - A digital bandpass filter with central frequency *f* is implemented.
  - Phase and quadrature components are obtained.
  - Reception beamforming for steering angle *θ_i_* is done.
  - Signal envelope is obtained.
  - Signal is filtered with a matched filter.
  - Signal is assembled in a two-dimensional array.

**Figure 4.**
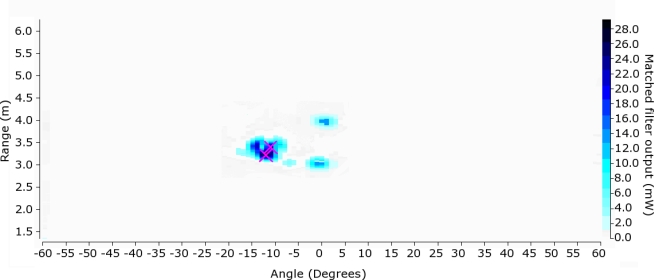
Acoustic image.

After processing *M* steering angles, there is a two-dimensional array that represents the acoustic image, as it is shown in Figure 4.

An application that runs, in a distributed way on two processing hardware platforms: PC and DSP, has been developed. The software that runs the PC has been developed in Visual C ++. And the software that runs the DSP has been developed in C++ and uses the “Malibu” DSP library from Innovative Integration.

**Figure 5.**
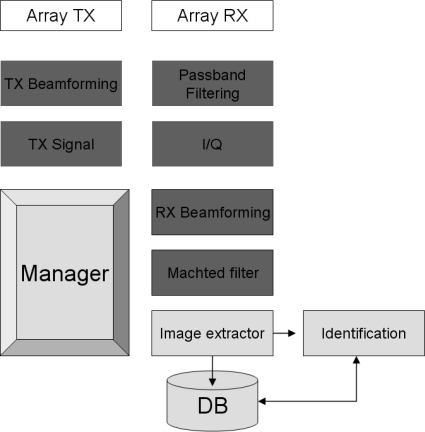
Functional distribution.

Acquisition, filtering and beamforming are implemented on the DSP, and management, storage of images, biometric algorithms and user interface are implemented on the PC. Figure 5 shows, in light gray, the functions implemented on the DSP and, in dark gray, the functions implemented on the PC.

The application software developed has four operation modes: Channel Calibration, Surveillance, Image acquisition and Biometric identification.

(1) Channel calibration

Experimental measurements have revealed that phase-shift errors and gain errors appear due to small differences in the electronic components of the analog processing chain. Beamforming requires that all channels must have the same phase and gain. Therefore a calibration procedure to compensate the gain errors and the phase-shift errors in each channel has been established [19]. The acoustic array uses a set of reference microphone and speaker in front of the array. The calibration algorithm uses the microphone to calibrate the speakers of the TX array and the speaker to calibrate the microphones of the RX array. Using the reciprocal sensor as a common reference, gain and phase-shift errors are calculated and applied in beamforming.

(2) Surveillance

In this mode, the system can detect and estimate the position of the targets present in the chamber, visualizing an acoustic image.

(3) Image acquisition

In this mode, the system captures the acoustic image of a person for a predefined set of frequencies and positions.

(4) Biometric identification

Previously, the system acquires the acoustic images for a set of *N* individuals, which are stored in a database.

Next, for the person under analysis, the system gets the acoustic images and compares them with the images stored in the database, performing the biometric identification.

### 2.2. Hardware Architecture

The biometric system has four elements:

- A computer with a real-time acquisition system for 16 channels.
- A preamplifier and amplifier system.
- A transmission array (TX array) and a reception array (RX array).
- An acoustic anechoic chamber.

**Figure 6.**
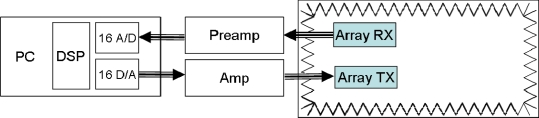
Block diagram.

Figure 6 shows a block diagram of the system and the interconnection between its elements.

#### 2.2.1. Personal Computer with Data Acquisition and Signal Processing Subsystems

**Figure 7.**
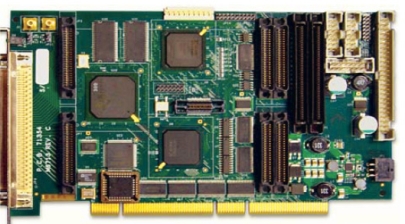
M6713 card.

These subsystems are based on a PC with a Pentium processor, which houses a Innovative Integration M6713 card, as shown in Figure 7, with a C6713@300MHz, 1.5 M gate FPGA Xilinx Spartan-3 DSP and two Omnibus I/O Daughter Card sites. This card is designed to work in the Radar, Sonar and Sodar fields.

**Figure 8.**
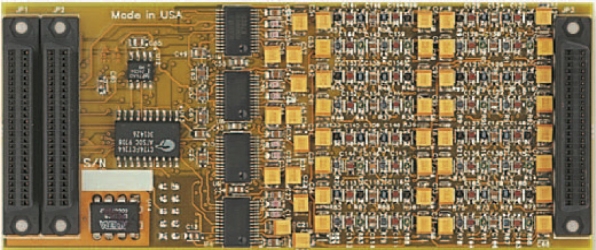
SD16 module.

M6713 hosts a module Omnibus SD16, shown in Figure 8, which provides 16 channels of 18 bit, 48 kHz sigma-delta A/D and D/A converters. Each input channel employs sigma-delta modulation with 384x oversampling, providing highly effective digital anti-alias filtering. The output channels include an 8X interpolation filter, digital attenuation and de-emphasis.

#### 2.2.2. Preamplifier and Amplifier Systems

**Figure 9.**
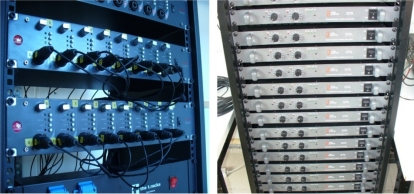
Preamplifiers and amplifiers.

This subsystem is formed by two SM PRO AUDIO EP84 multichannel microphone preamplifiers and eight T. AMP S75 amplifiers, installed on a 19” rack with 48U, as shown in Figure 9.

**Figure 10.**
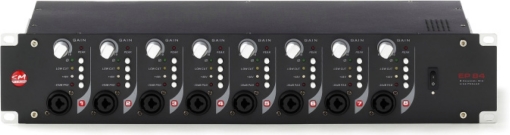
Microphone preamplifier SM PRO AUDIO EP84.

The SM PRO AUDIO EP84 preamplifier features eight channels, independent variable gain control, −60 dB microphone sensitivity, XLR and TRS input and output, 48V Phantom power per channel, 20 Hz–20 kHz bandwidth, −20dB pad, phase reverse and low cut filters. The EP84 is shown in Figure 10.

**Figure 11.**
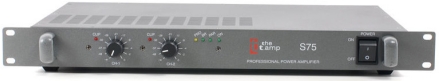
Amplifier T.AMP S75.

The T.AMP S75 amplifier, shown in Figure 11, features two channels, 75W/4Ohm, soft start and balanced XLR & jack.

#### 2.2.3. Transmitter and Receiver Arrays

**Figure 12.**
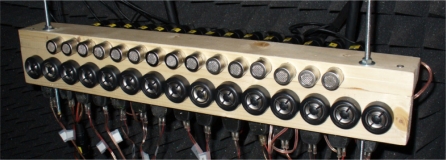
Transmitter and receiver arrays.

This subsystem consists of a receiver uniform linear array (ULA) with 15 BEHRINGER C2 studio microphones with omnidirectional pattern and a ULA transmitter with 15 HPC tweeters, as shown in Figure 12.

#### 2.2.4. Acoustic Anechoic Chamber

**Figure 13.**
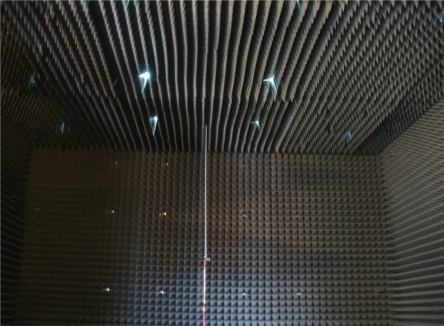
Acoustic anechoic chamber.

The acoustic anechoic chamber has a 5 × 3 × 2.5 m working area. To eliminate reflections from the walls of the chamber, every surface is covered with absorbent materials with 50 × 50 × 170 mm foam pyramidal wedges. The chamber has been designed for a 500 Hz cut-off frequency. This chamber is shown in Figure 13.

## 3. Acoustic Array Sensor

### 3.1. Spatial Aperture Selection

In the design process of the spatial aperture (length) of transmission and reception ULAs, the following parameters must be considered: angular resolution, frequency band, angular excursion and transducer diameter. If the array spatial aperture is increased, the angular resolution improves, however grating lobes appear [16].

Two ULAs with 15 λ/2-equispaced sensors have been employed. These arrays have different spatial apertures in order to reduce sidelobe levels on the final beampattern (Tx + Rx). Note that sidelobe positions on each beampattern are different, while the mainlobe keeps its position.

A transmission array with a 50 cm spatial aperture and a reception array with a 40 cm spatial aperture has been used. On the transmission array, the tweeters are placed so as to occupy the minimum space.

### 3.2. Frequency Band Selection

After defining the array spatial apertures, it is necessary to evaluate the range of frequencies where the array works properly. This evaluation is based on:

- The angular resolution: 3-dB beamwidth of the mainlobe.
- Non appearance of grating lobes.
- Frequency response of the microphone-tweeter pair.
- Frequency response of a person.

Working with low frequencies increases the main beam width and therefore degrades the angular resolution. Working with high frequencies decreases the main beam width, but grating lobes appear, which degrades the beampattern. On the other hand, we note that main lobe width and grating lobes level increases as the steering angle rises. Therefore, the maximum steering angle should be determined by the size of the person and his/her distance from the array.

Based on these considerations, the following parameters have been selected:

- The positioning area is located 3 m from the array
- The maximum width of a person with outstretched arms is 2 m
- The range is 2.5 m

**Figure 14.**
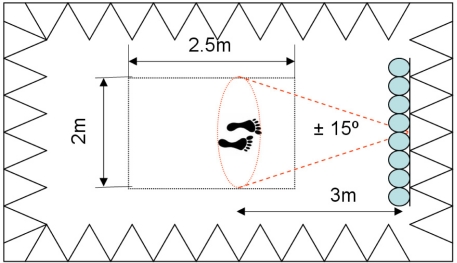
Scanning and positioning area.

For these parameters, the angle excursion is ±15°, as shown in Figure 14.

Then, the beampattern for the broadside (*θ* = 0°) and maximum steering angle (*θ* = 15°) have been analyzed, with a sweeping frequency from 4 to 16 kHz in intervals of 1 kHz. The lower and upper frequencies where the main lobe width and the grating lobes level are appropriate for the system have been determined, obtaining the values 4 kHz and 14 kHz, respectively.

**Figure 15.**
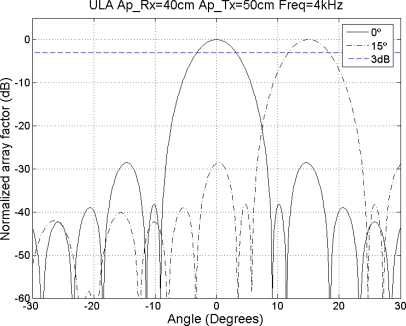
Beampattern for 0° and 15° for *f* = 4 kHz.

Figure 15 shows the beampattern of the array proposed for *θ* = 0° and *θ* = 15° for the lower working frequency, *i.e.*, 4 kHz. It can be observed that there are no gratings lobes and the main lobe width range from 6.4° on the broadside to 6.7° on the maximum steering angle.

**Figure 16.**
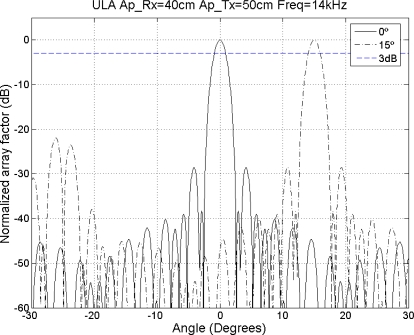
Beampattern for 0° and 15° for *f* = 14kHz.

Finally, Figure 16 shows the beampattern of the proposed array for *θ* = 0° and *θ* = 15° for the higher working frequency. *i.e.*, 14 kHz. It can be observed that there are no gratings lobes and main lobe width ranges from 1.80° on the broadside to 1.85° on maximum steering angle.

**Figure 17.**
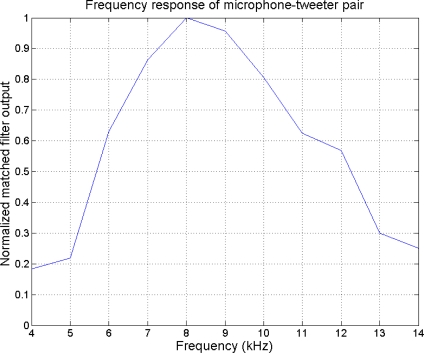
Frequency response of microphone-tweeter.

For frequencies below 4 kHz, or above 14 kHz the beampattern degrades significantly and cannot be used. Analyzing the frequency response for the microphone-tweeter pair used (Figure 17), the following results have been obtained.

For frequencies below 6 kHz and for frequencies above 12 kHz, the system sensitivity is very low, due to the pass-band response of the tweeter. Therefore a frequency band between 6 kHz and 12 kHz has been selected.

At this point, the number of frequencies and values must be determined. It is clear that a large number of frequencies allow people characterization, but at the expense of increasing acquisition and processing times. On the other hand, a high number of frequencies does not improve the system performance, as the frequencies have to be closer and the obtained images are not independent.

After several tests, four different frequencies that guarantee the independence of the obtained images have been selected. The determination of the optimal values would be a very complex process, because it would depend on people are wearing and an exhaustive study will be required. Finally, the selected frequencies are 6 kHz, 8 kHz, 10 kHz and 12 kHz, where the frequency gap is the maximum in order to have independent images.

### 3.3. Angle Resolution Cells and Number of Beams

Given a ULA, Δ*u* is defined as 3-dB beamwidth of the mainlobe in the *sin(θ)* space. Beamwidth in *sin(θ)* space does not depend on the steering angle and therefore, assuming that beams are 3-dB overlapped, the number of beams necessary to cover the exploration zone will be [16]:

(1) $$M=\mathrm{round}\left(\frac{2\cdot \text{sin}\;\theta_{\text{max}}}{\Delta u}\right)$$

where *θ_max_* is the angular excursion.

After evaluating the final beampattern of the transmission and reception arrays, Δ*θ* is obtained. Δ*θ* is defined as the 3-dB beamwidth of the mainlobe in degrees. Then, beamwidth in the *sin(θ)* space is obtained using the expression:

(2) Δu=sinΔθ

Finally, the number of beams for each frecuency, *M_k_*, is calculated using Expressions (1) and (2). These values are shown in Table 1. A value of *θ_max_* = 15° for the angular excursion has been assumed.

**Figure 18.**
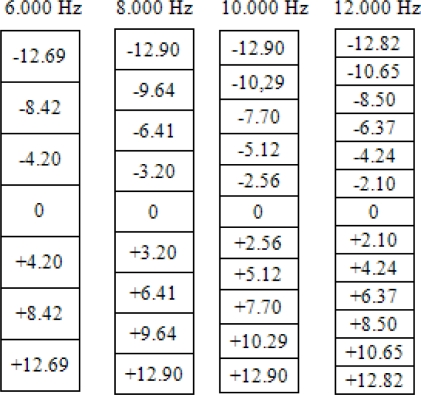
Steering angles *vs.* frequency.

Steering angles for each frequency are shown in Figure 18.

**Figure 19.**
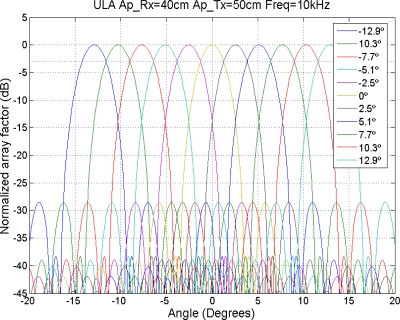
Collection of beampatterns to cover the exploration zone for *f* = 10 kHz.

Finally, the collection of beampatterns necessary to cover the exploration zone for *f* = 10 kHz is shown in Figure 19.

## 4. Definition and Extraction of Acoustic Profiles

A collection of samples of acoustic images, with the procedures and parameter values used for image acquisition and identification are presented in this section.

### 4.1. Image Parameters

Following the design considerations of Section 3, the system retrieves the acoustic image associated to a rectangle of 2 m × 2.5 m (width × depth) dimensions, where the person under analysis must be located 3 meters away from the line array, as described in Figure 14. As justified in the previous section, 4 frequencies: *f_1_* = 6 kHz, *f_2_* = 8 kHz, *f_3_* = 10 kHz and *f_4_* = 12 kHz were selected.

A 2 ms pulse width value has been selected. This value is a trade-off between range resolution, which is inversely proportional, and the received energy, which is proportional. The acoustic images are collected from 2.0 m to 4.5 m, in range coordinate, and from −15° to 15°, in azimuth coordinate, using *M_i_* steering angles. The acoustic images are stored into a matrix *I*:

(3) $$I\left(n,m\right)\{\begin{array}{cc} 1\leq n\leq N & \mathrm{range}\\ 1\leq m\leq M_{k} & \mathrm{azimuth} \end{array}$$

Assuming a sampling frequency *f_s_* = 32 kHz and a sound velocity *v* = 340 m/s, the matrix dimension *N* will be:

(4) N=2.5m⋅32 kHz/340=235

The matrix dimension *M_k_* is the number of steering angles necessary to cover the exploration area for each frequency.

### 4.2. Positions

**Figure 20.**
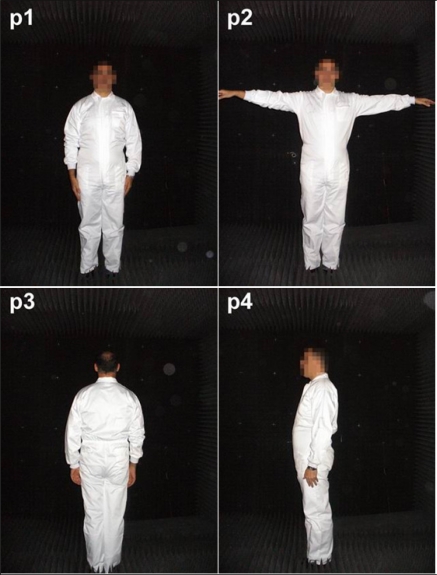
Person positions.

After analyzing multiple positions of people was concluded, it has been determined that the best results are obtained for the following positions: front view with arms folded on both sides (*p_1_*), front view with arms outstretched (*p_2_*), back view (*p_3_*) and Side view (*p_4_*). Figure 20 shows the four positions using a test subject.

These positions are associated with the different body sections and therefore are clearly differentiated. These positions are highly independent, improving system biometrics performance. Only these four positions have been used because a higher number would increase acquisition and processing times, without a noticeable improvement of the biometric system performance.

### 4.3. Acoustic Profile

The acoustic profile *P_i_*, associated to person i, includes the acoustic images obtained for the positions (*p_1_*, *p_2_*, *p_3_*, *p_4_*), evaluated at frequencies (*f_1_*, *f_2_*, *f_3_*, *f_4_*).

**Figure 21.**
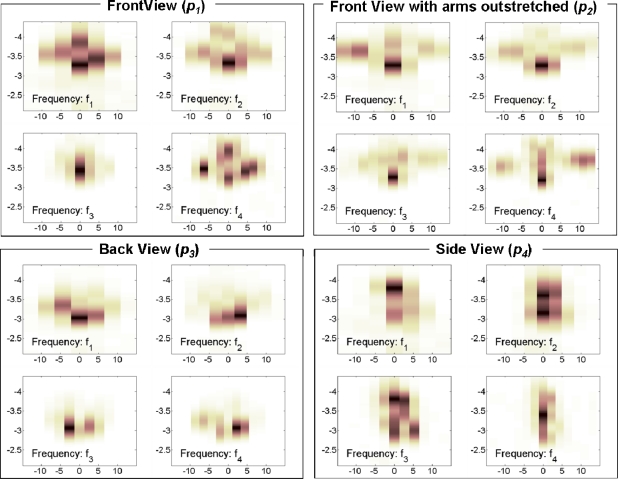
Acoustic images. x-axis: angle (degrees), y-axis: range (m).

Figure 21 shows the acoustic images for: i) the front view position (*p_1_*), where the head and trunk of the subject can be clearly identified ii) the front view position with arms outstretched (*p_2_*), where the head and arms of the subject can be clearly identified, iii) the back view position (*p_3_*) where the back of the head can be identified and iv) the side view position (*p_4_*), where the closest shoulder and side of the head can be identified.

### 4.4. Image Normalization

Each acoustic image is normalized for its energy, according to the expression:

(5) $$\bar{I}\left(i,j\right)=\frac{I\left(i,j\right)}{\sqrt{\sum_{n=1}^{N}\sum_{m=1}^{M_{k}}I{\left(n,m\right)}^{2}}}$$

## 5. Test Acoustic Profiles for Biometric Applications

### 5.1. Metric Based on Mean Square Error

An algorithm for biometric identification has been implemented based on the mean square error between acoustic images from the profile *P_i_* and the profile *P_j_* [20]. First, the function $E_{p}^{f}\left[i,j\right]$ is defined as the mean square error between an acoustic image from profile *P_i_* and an acoustic image from profile *P_j_*, for a specific frequency *f* and a position *p*:

(6) $$E_{p}^{f}\left[i,j\right]={\sum_{n=1}^{N}\sum_{m=1}^{M}\left({\bar{I}}_{i}\left(n,m\right)-{\bar{I}}_{j}\left(n,m\right)\right)}^{2}\;i,j=1\ldots NF$$

where *NF* is the number of acoustic profiles stored in the database.

Then, the function *E_p_[i,j]*, called multifrequency error, is defined as the sum of the errors at each frequency for the position *p*:

(7) $$E_{p}\left[i,j\right]=E_{p}^{6\mathrm{kHz}}\left[i,j\right]+E_{p}^{8\mathrm{kHz}}\left[i,j\right]+E_{p}^{10\mathrm{kHz}}\left[i,j\right]+E_{p}^{12\mathrm{kHz}}\left[i,j\right]$$

Finally, the function *E(i,j),* called global error, is defined as the sum of the multifrequency error at each position *p*:

(8) $$E[i,j]=E_{p1}\left[i,j\right]+E_{p2}\left[i,j\right]+E_{p3}\left[i,j\right]+E_{p4}\left[i,j\right]$$

If *P_k_* is an unknown profile to be identified, the algorithm will associate the profile *P_k_*, to the person “*i*” in the database, whose profile *P_i_* has the minimum *E[k,i]* value. The normalized global error will be defined as the distance or metric used by the biometric system.

### 5.2. Scenario Definition

The case-study involved 10 people–5 men and 5 women–in order to analyze the behaviour of the system. Each selected person has distinct morphological features, as shown in Table 2. In this analysis, all people use an overall as common reference clothing, in order to eliminate clothing as a distinctive factor. The biometric system uses a metric or distance based on the mean square error, according to expression (6).

To evaluate the system, acoustic profiles were captured 10 times for each of the 10 people under test. These captures were carried out for 10 days, one capture per person per day, thereby people did not remember their position in the previous capture and there was not a “memory effect”. They were placed in the center of the measurement area (cross marked on the ground). Every 60 seconds, a multifrequency capture was done for each position, with the following sequence: front view (*p_1_*) with arms folded on both sides, front view with arms outstretched (*p_2_*), back view (*p_3_*) and side view (*p_4_*).

One hundred profiles were acquired; each one with 16 acoustic images (four frequencies by four positions). These captures were stored with a unique identifier formed by sub identifiers associated with the person ID, the number of capture, the position and the frequency. Finally, the normalized global error between all acquired profiles was calculated.

### 5.3. False Match Rate (FMR) and False Non-Match Rate (FNMR)

Based on the methodology to characterize a biometric system [21], FNMR and FMR parameters have been calculated. It is assumed that there are no errors in the acquisition; therefore FAR/FMR and FRR/FNMR pairs are equivalent.

False match rate (FMR) is the probability of the system matching incorrectly the input acoustic profile to a non-matching template in the database, i.e. the percentage of imposters incorrectly matched to a valid user’s biometric. It measures the percent of invalid inputs which are incorrectly accepted. FMR is obtained by matching acoustic profiles of different people. The global error *E(i,j)* is calculated for all these cases; then the FMR parameter is calculated as the percentage of matching whose error value is equal or less than the distance *d*:

(9) E(i,j)≤d

Where the distance *d* is the set of possible values of the global error. False non-match rate (FNMR) is the probability of the system not matching the input acoustic profile to a matching template in the database, i.e. the percentage of incorrectly rejected valid users. It measures the percent of valid inputs which are incorrectly rejected. FNMR is obtained by matching acoustic profiles of the same people. Again, the normalized global error is calculated for all these cases; then the FNMR parameter is calculated as the percentage of matching whose error value is greater or equal than the distance *d*:

(10) E(i,j)≥d

**Figure 22.**
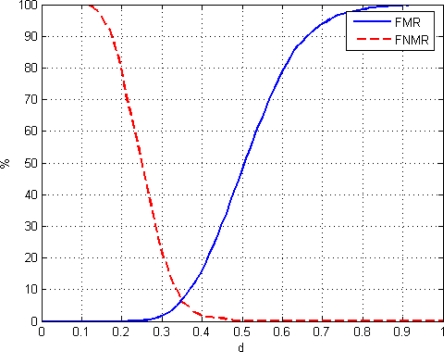
Functions FMR and FNMR *vs.* distance *d*.

Figure 22 shows the FMR and the FNMR functions versus the normalized distance *d*.

### 5.4. Equal Error Rate (EER)

**Figure 23.**
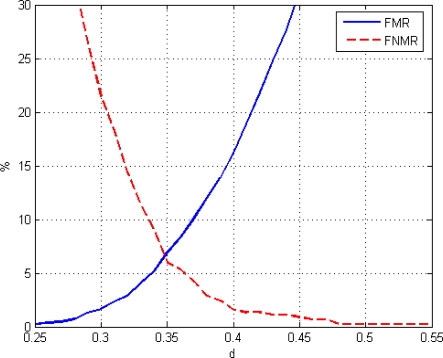
EER.

Equal Error Rate (EER) is the crossing point between FMR and FNMR functions. EER is a global parameter that allows the evaluation of the system performance. Thus, the lower the EER value, the better the biometric system performance [21]. For this case-study, a value of EER = 6.53% for a distance *d* = 0.35 has been obtained. Figure 23 shows an enlarged area of FMR and FNMR functions in order to show EER clearly.

### 5.5. Receiver Operating Characteristic (ROC)

**Figure 24.**
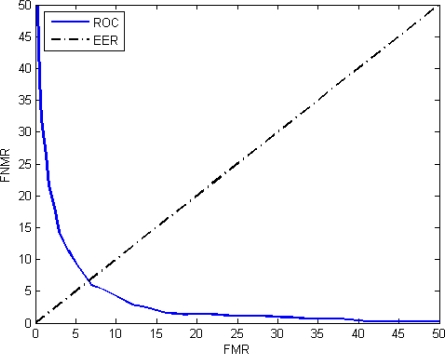
ROC.

Finally, the ROC curve, a visual characterization of the trade-off between the FNMR and the FMR, is shown in Figure 24.

Besides this system, many other novel biometric systems expect to give an alternative to the well known biometric systems based on fingerprints. There are analyses of systems based on hand geometry [22], on finger geometry [23], on finger veins [24] or even on the mouse dynamics [25], amongst others.

Comparing the results, particularly the EER value, obtained in the study case shown in this work with the results shown by other novel biometric systems, it can be observed that the EER value of the system based on acoustic images is comparable to the behaviour of other novel biometric systems. Table 3 shows the EER values of different novel biometric values.

Results in Table 3 are quite promising and confirm the feasibility of using acoustic images in biometric systems. The results obtained in this work are based on the mean square error, which is a robust and simple metric. As more complex metrics are developed, the system performance will be better.

## 6. Conclusions

An acoustic biometric system based on an electronic scanning array using sound detection and ranging techniques has been developed. People are scanned with a narrow acoustic beam in an anechoic chamber, and then an acoustic image is created by collecting people's response to the transmitted signal.

This work is focused on analyzing the feasibility of employing acoustic images of a person as a biometric feature. Specifically, four pulsed tone signals with frequency 6 kHz, 8 kHz, 10 kHz and 12 kHz and four positions for the person (front, front with arms outstretched, back and side) have been used, getting a representative set of acoustic images.

FNMR, FMR and the ROC curve have been obtained, being comparable to those of commercial biometric systems. These facts confirm the feasibility of using acoustic images in biometric systems. Currently, work on improving algorithms and extending the case-study presented with a broader set of users is being carried out. The weights of the different acoustic images (frequency and position) in the error function are also being optimized.

## Figures and Tables

**Table 1.** Number of beams *vs.* frequency.

**F (Hz)**	Δ*θ***(degrees)**	Δ*μ*	**M_k_**
6000	4.20°	0.0732	7
8000	3.20°	0.0558	9
10000	2.56°	0.0447	11
12000	2.12°	0.0370	13

**Table 2.** Morphological features.

**Properties**			
**ID**	**Gender**	**Constitution**	**Height**
00	Male	Very strong	Tall
01	Male	strong	Average
02	Male	strong	Average
03	Male	Thin	Tall
04	Male	normal	Tall
05	Female	Thin	Tall
06	Female	strong	Small
07	Female	Thin	Average
08	Female	strong	Average
09	Female	normal	Small

**Table 3.** EER of novel biometric systems

**Novel biometric system**	**EER**
Acoustic images	6.53%
Hand geometry	7.7–13.2%
Finger geometry	17.86–23.16%
Finger vein	1.91–14.8%
Mouse dynamics	3.8%
